# Community dimensions and emotions in the era of COVID‐19

**DOI:** 10.1002/casp.2560

**Published:** 2021-08-15

**Authors:** Daniela Marzana, Cinzia Novara, Norma De Piccoli, Paola Cardinali, Laura Migliorini, Immacolata Di Napoli, Elisa Guidi, Angela Fedi, Chiara Rollero, Barbara Agueli, Ciro Esposito, Elena Marta, Florencia González Leone, Andrea Guazzini, Patrizia Meringolo, Caterina Arcidiacono, Fortuna Procentese

**Affiliations:** ^1^ Department of Psychology Università Cattolica del Sacro Cuore and CERISVICO Research Centre on Community Development and Organisational Quality of Life Brescia Italy; ^2^ Department of Psychology, Educational Science and Human Movement University of Palermo Palermo Italy; ^3^ Department of Psychology University of Torino Torino Italy; ^4^ Department of Education Science University of Genoa Genova Italy; ^5^ Department of Humanities University of Naples Federico II Napoli Italy; ^6^ Department of Education, Languages, Intercultural studies, Literatures and Psychology, and Center for the Study of Complex Dynamics (CSDC) University of Florence Florence Italy

**Keywords:** anxiety, connectedness, emergency, emotional sharing, solidarity

## Abstract

Following an ecological perspective, reactions to a disaster—such as the COVID‐19 pandemic—should be analysed in the interdependence between individual and community dimensions. The present study aims to analyse individual emotional dimensions (anxiety, joy, fear or depressive feelings) and their community dimensions (connectedness, emotional sharing and solidarity) with a longitudinal approach among university students from Italian universities. Participants were 746 university students at t1 (during the lockdown) and 361 at t2 (after the lockdown) recruited in six Italian universities from different areas of Italy. Comparing emotional dimensions in the two times, t2 is characterized by a generalized ambiguity: both happiness or joy because of the end of limitations and a kind of ‘post‐lockdown anxiety’ because of a sense of individual inadequacy in facing the return to normality, conducting daily activities and attending community spaces. Data confirms that after the so‐called ‘honeymoon phase’ in community dimensions (first phase of t1 time), a sort of ‘depressive reaction’ arises at t2: Italian university students seem more aware of the need for individual and social responsibility and that many events are not under their personal control. The reconstruction phase and exit from the emergency are perceived as necessary but also as a difficult and risky period. Please refer to the Supplementary Material section to find this article's Community and Social Impact Statement.

## 1 INTRODUCTION

The pandemic caused by COVID‐19 is an exceptional event that calls into question both individuals and entire communities committed to finding resources and strategies to cope with the contingent situation and future prospects. This means that both lifestyles and social relationships are also changing throughout the world.

There have been and continue to be many effects that have completely new features compared to those known to date. In fact, the COVID‐19 pandemic is the largest atypical pneumonia since the outbreak of Severe Acute Respiratory Syndrome (SARS) in 2003 and had already affected the former outbreak's total number of confirmed cases in the first weeks of the epidemic; moreover, related deaths have exceeded those of SARS (Wang et al., 2020), with a human‐to‐human transmission of the COVID‐19 virus (Huang & Zhao, 2020) that quickly spread to the global community (Fardin, 2020). In a short time, the impact on the whole community required swift containment measures that entailed social isolation; furthermore, continuous changes related to the evolution of the pandemic and related containment measures produced negative emotions (Blendon, Benson, DesRoches, Raleigh, & Taylor‐Clark, 2004; Pfefferbaum & North, 2020). In a complex event such as the COVID‐19 outbreak, there were many people involved and a strong emotional impact. In order to express these human experiences, emotions centred in subjective experiences were represented by language (Barrett, Lindquist, & Gendron, 2007); in fact, people represent their experiences within a semantic space that includes many terms that refer to a rich variety of emotional states (Rüssel, 1991) most influenced by the situations in which they occur (Clore & Ortony, 2013; LeDoux & Brown, 2017).

The novelty of this study is in the analysis of individual narratives aimed at collecting not only emotions and moods related to individual well‐being, but also feelings and actions connected to significant others as well as to the whole community of belonging. Specifically, all individual and community dimensions were situated in the Italian specific context: that is, they were framed by the incidence and prevalence of the virus as well as by the public measures of contrast and support.

The COVID‐19 pandemic and the correlated restrictions have had a tragic impact on the lives of people and communities; for this reason, it becomes important to understand individual emotional responses and their community dimensions with respect to this devastating event. The analysis of these aspects is central to detecting the impact that COVID‐19 has had on people and to formulating guidance for interventions (Kleinberg, van der Vegt, & Mozes, 2020).

This pandemic has increased anxiety and depression (Fardin, 2020), making people more susceptible to psychological distress. The strongest predictor of behaviour is the perception of severity, followed by confusion about the reliability of the available information (Qian et al., 2020).

People, in fact, deployed creativity and resources to feed the sense of connectedness, emotional sharing and solidarity, thus counteracting negative emotions and the uncertainty of the moment (Brooks et al., 2020; Van Bavel et al., 2020; Vergin, 2020). Specifically, participation and the sense of collective responsibility became tools to contain the emergency (Procentese & Gatti, 2019).

Indeed, knowledge, perception, preventive behaviours and social participation of the population are important for controlling the epidemic (Procentese, Capone, Caso, Donizzetti, & Gatti, 2020).

Community psychology is an approach devoted to the study of people's voices, their interactions and actions for promoting social change supporting their aims. In fact, community psychology is at the boundary between individuals and their contextual environment as Lewin (1936) highlighted, (Orford, 2008) reminded us and, in Italy, was widely discussed by Amerio (2000). In this connection, facing the threat of this unprecedented pandemic to one's own and others' health as well as the effects of home confinement on individual well‐being have become a pressing concern. In fact, each individual is in a mutual relationship of influence and dependence with the context in which he/she lives (Maton, Perkins, & Saegert, 2006).

Evaluating the effects of the current pandemic by the assumptions of community psychology means assuming as a unit of analysis ‘the person in context’; that is, paying attention to both the individual's emotional response to the crisis situation and the community dimensions that express the way in which people organize themselves to give common and shared answers to problems (Maya Jariego, 2016; Trickett, 1995).

Individual and collective responses are largely influenced by contextual experiences: for example, during lockdowns following the pandemic, the restrictive measures were regulated by law and have to be followed by everyone; after the lockdown, people display greater individual decision power and a less unanimous adherence to the rules (Prosser, Judge, Bolderdijk, Blackwood, & Kurz, 2020). Therefore, this article examines individual emotional responses and their community dimensions during t1 of lockdown and in the transition to t2 of post‐lockdown, focusing on university students.

### 1.1 The pandemic as an emergency

The COVID‐19 pandemic, due to its implications on multiple levels, can be considered a cascading emergency (Pescaroli & Kelman, 2017). In fact, the COVID‐19 pandemic has generated a health and economic crisis and is inscribed in the social context as in other natural disasters (e.g., fires and floods) and social challenges (e.g., inequality, racism and poverty). Like any emergency, the current one is also characterized by different phases in which people act in the individual and social spheres in different ways.

The Centre of Mental Health Services distinguishes four phases of disasters, based on the emotional response of people who have been involved in an emergency situation: *Heroic phase*, in which strong emotions appear and people are forced to carry out heroic actions; *Honeymoon phase*, in which there is a strong feeling of community sharing about a difficult experience and there is a common idea of being able to overcome disaster; *Phase of Disillusionment*, in which the emotions of anger and resentment prevail and in which the sense of community sharing is gradually lost, and people start to focus on personal problems in order to return to normality and to rebuild the reference points of their existence; *Reconstruction phase*, in which the awareness of having to personally take charge of the resolution of problems gradually emerges.

However, the current emergency has dragged on with an impact that fuels collective and individual discomfort and illness as well as difficulties in creating ever new meanings and adaptive responses (Weick, 2020). Therefore, the phases describing the effects of disasters are only partially adequate to explain this historical moment because the disillusionment phase is not yet fully visible due to the recursive nature of the present pandemic. Coronavirus, like other emergencies, makes people feel that they are under attack by a common and indiscriminate enemy (Walker, 2020).

### 1.2 Individual emotional dimensions and community dimensions

Emotions include a cognitive evaluation (of both internal and external factors) and synthesize a complex experience of multi‐componential responses that, in turn, disposes people to act in one direction or in a completely opposite one (Arnold, 1960). Emotions, then, allow people to exert a certain control over events; meanwhile, the regulation mechanisms themselves are part of the emotional experience. In emergency emotions act as the driving force of action, influencing personal well‐being and one's relationship with the community. In the COVID‐19 pandemic, many people have been implicated on the emotional level with strong emotions like fear, worry, stress, anxiety and depression. Many studies, in different geographical contexts, demonstrated empirical evidence in support of this specific emotional spectrum, including university students (Capone, Caso, Donizzetti, & Procentese, 2020). Most of these studies described students' emotional responses caused by their confinement at home, exploring the influence of their household organization (living with parents), social support or also direct experience of COVID (by the student or significant others) (Aristovnik, Keržič, Ravšelj, Tomaževič, & Umek, 2020; Aylie, Mekonen, & Mekuria, 2020; Pagnini et al., 2020; Pragholapati, 2020; Son et al., 2020). The focus only on individual emotions is hardly sufficient because it misses the crucial dimension of the community and its impact on the emotions experienced by individuals; therefore, our reference are the individual emotional dimensions, but the community psychology lens makes us deeply concerned with issues of feelings, action and participation relating to the relational and community context.

## 2 PRESENT STUDY

The general purpose of the study was to explore the behaviours, feelings, thoughts and actions of university students during the lockdown and immediately after. The two dimensions on which we focused are (a) the individual emotional dimension, experienced over time in changing conditions, and (b) community dimensions such as connectedness, emotional sharing and solidarity, which emerge in emergency situations such as that produced by COVID‐19.

This research was carried out in two times: during the first lockdown (March, 2020) and during the following country's reopening time (May, 2020). The choice to focus on students was dictated by the observation that this group is among the most exposed to the pandemic risks. Saita, Facchin, Pagnini, & Molgora, (2021) found that students were more distressed than other groups with higher anxiety, depression, and perceived stress. In this regard, the uncertainty related to the sudden, unexpected transition to distance learning, and concerns about the future (Sahu, 2020) might have played an important role, with negative effects on the psychological health of this younger subgroup of people (Saita et al., 2021).

Six Italian universities undertook a project on the effects of the COVID lockdown in the Italian population of university students. In order to describe the relations existing between context and the individual dimension, we included in the study students from various cities consistent with the prevalence of COVID‐19 in those places to ensure a certain national representativeness. During the first lockdown (from March to May 2020), Italy was divided into three zones (red, orange and green), corresponding to the contagion risk levels of the COVID 19 pandemic in the regions of the country. The red zone is for the regions with the highest risk, orange for those with an intermediate risk, and green for the lowest risk. Thus, for the ‘Red zone’, we selected the provinces of Milan and Turin, located in the northern regions where the highest level of contagion was reported. Florence and Genoa represented the ‘Orange zone’, with a medium level of contagion. Finally, the ‘Green zone’ included provinces of Naples and Palermo, where contagion was very low. In the context of this research, a grounded theory methodology (GTM) was used as an interpretative approach; this method of analysis was chosen because it is suited to explore a phenomenon in the absence of pre‐defined specific hypotheses about the issues to investigate. As reported by Rasmussen et al. (2016), grounded theory ‘at the most basic level … remains an approach in which researchers use data to develop theory from the bottom up’ (p. 23). The research team was composed of university instructors and researchers in community psychology who address individual and collective well‐being in their regional communities with particular reference to emotional connection and sense of belonging. A focalized approach to storytelling was proposed as a useful tool to facilitate and share a self‐reflection process on the experience of the pandemic situation. In fact, we asked students to write down their feelings (including those specific to their personal context), thoughts and action related to this unprecedented experience, with a maximum of 5,000 characters. We gave them an open‐ended prompt to describe this unexpected and sudden experience. We did not provide a grid, nor ask for specific content or provide specific items.

## 3 METHOD

### 3.1 Participants and procedure

Participants at t1 (data collected in March 2020) were 746 (118 males and 628 females), with a mean age of 21.93 years (*SD* = 4.27). Participants were recruited by convenience sampling facilitated by the presentation of the project during the classes of Community Psychology. The teachers presented the research project to the students during the lessons. Later an email was sent to them with the link to connect to in order to fill in the questions anonymously.

To carry out the t2, the same students were contacted through the e‐mail addresses or mobile phone numbers they had left as data in the first time.

In this second data collection (May 2020), 361 participants described their experience again. Of these, 60 were males and 301 females, and the average age in this case was 21.91 years (*SD* = 4.72).

In Table 1, all the characteristics of the participants are synthesized.

**TABLE 1:** Characteristics of the participants in the two waves

	First wave	Second wave
	*n* = 746	*n* = 361
	*M* = 21.93 (*SD* = 4.27)	*M* = 21.91 (*SD* = 4.72)
*Age*	*N* (%)	*N* (%)
*Sex*		
Male	118 (15.8%)	60 (16.6%)
Female	628 (84.2%)	301 (83.4%)
*Territorial area*		
North (Catholic University of Milan, University of Turin)	328 (44.0%)	95 (26.3%)
Centre (University of Florence, University of Genoa)	66 (8.8%)	32 (8.9%)
South (University of Naples Federico II, University of Palermo)	352 (47.2%)	234 (64.8%)
*Level of the course of study*		
Bachelor's degree	597 (80.0%)	334 (92.5%)
Master's degree	149 (20.0%)	27 (7.5%)
*Context of origin*		
Urban area	545 (73.9%)	249 (69.0%)
Rural area	201 (26.1%)	112 (31.0%)
*Housing condition*		
With one or both parents	631 (84.6%)	307 (85.0%)
Alone	20 (2.7%)	9 (2.5%)
With the partner	33 (4.4%)	16 (4.4%)
With one or more roommates	26 (3.5%)	10 (2.8%)
With other family members	36 (4.8%)	19 (5.3%)

### 3.2 Data analysis

The textual materials we collected were analysed by means of GTM (Charmaz & Belgrave, 2019; Corbin & Strauss, 2008) using ATLAS.ti 8.4.

As defined by GTM, a bottom‐up approach was followed and the procedure codes developed over three phases of the abstraction process: open coding, axial and selective coding. Our data processing was carried out in keeping with the standards for reporting qualitative research (SRQR) (O'Brien et al., 2014), which gives operational guidelines for doing and writing about qualitative research as well as data processing methods.

## 4 RESULTS

The analytical process led to the identification, within the two macro‐categories of the individual emotional dimensions and the community dimensions, of seven sub‐categories for the individual emotional dimensions and three sub‐categories for the community dimensions. Each sub‐category collected a certain number of codes. A descriptive picture of this complex coding process is in Table 2, which reports the codes used.

**TABLE 2:** Codes and categories

Codes	Sub‐categories	Macro‐categories
Concern	Concern	Individual dimensions
Concern related to loved ones		
Concern related to transgression of the rules		
Excessive concern for one's state of health		
Concern about the risk of invalidating sacrifices		
Apprehension		
Tension		
Turmoil		
Fear	Fear	
Fear for a new increase in infections		
Fear for the return of the lockdown		
Fear of getting sick		
Fear of returning to normality		
Fear of leaving the house		
Fear of being fine in isolation		
Fear of infecting		
Contrasting emotions: Fear mixed with joy, insecurity and awareness		
Anger	Anger	
Anger against people who do not respect the rules		
Anger towards local and national institutions		
Anger towards the imposed restrictive measures		
Anger due to feeling powerless		
Impotence	Depressive feelings	
Nostalgia		
Loneliness		
Sadness		
Apathy		
Feeling alone with oneself		
Contrasting emotions: Feeling discouraged, but at the same time hoping for the future		
Joy for a partial return to normality	Joy	
Joy for the chance to finally reunite with family and friends		
Joy to go out in the open air for walks or physical activity		
Joy for the chance to return to own workplaces		
Joy as an enhancement of rediscovery of important things		
Anxiety	Anxiety	
Difficulties in falling asleep		
Difficulties in studying		
Anxiety about the failure to return to normal life		
Anxiety for the uncertainty of the future		
Anxiety about the need for reorganization of one's own life		
Anxiety about returning to normality	Post‐lockdown anxiety	
Anxiety about exiting the house		
Sharing of remote activities	Emotional sharing	Community dimension
Sharing of the same contagion risk situation		
Flash mob		
Sharing of collective emotions		
Sharing of a common destiny		
Sharing of daily moments		
Altruism	Connectedness	
Sense of cohesion		
Sing hymns		
Sense of belonging		
Sense of community		
Collective support		
Need for social capital		
Mutual beneficial actions	Solidarity	
Beneficial actions by famous people		
Collective solidarity with the weakest		
Collective solidarity, towards and from health personnel		
Appreciation of volunteering		

### 4.1 Individual emotional dimensions

Emotions taken into consideration are concern, fear, anger, depressive feelings, joy and anxiety, with reference to post‐lockdown anxiety, a specific form of anxiety arising from the return to partial normality.

#### 4.1.1 Concern

The first period of lockdown brought a sort of overwhelming fear (see next paragraph) followed by concern related to loved ones and to transgression of the rules.

Meanwhile at t2, some forms of previous concern decreased, but there was an increase in excessive concern for one's state of health: ‘*Fear towards the people I meet lives on*, *due to the fear of being infected*, *but which I cannot control*, *leading to panic attacks or suddenly dropping out of conversations*, *followed by texted apologies*’ (M, 21, Naples, t2).

In addition, there was a new concern about the risk of invalidating sacrifices: ‘*Many of my peers did not understand the gravity of the situation and live as if nothing had happened in these two months*. *They would make all our sacrifices to have been in vain*’ (F, 22, Palermo).

There were, however, references to new emotions that were less intense but still had negative connotations such as apprehension, tension and turmoil.

#### 4.1.2 Fear

Fear characterized the first impact of the epidemic, and at the t2, there was indeed a strong reduction of this emotion compared to the t1.

However, at t2, the interviewees had different positions about fear: on the one hand, there were those who fear for the increase in infections and for the return of the lockdown while, on the other hand, there were those who have less fear of the virus: ‘*I thought that people would be more fearful*, *but there are really a lot of people out and about even with just the first easing of lockdown*’ (F, 22, Genoa, t2).

In particular, the fear of returning to normality, the fear of leaving the house and the paradoxical fear of feeling fine in isolation, that is, of not being able to feel good together with others, emerged as new emotions: ‘*At the same time I realize that when I am away from home*, *I live in a state of constant alert*’ (F 21, Florence, t2).

However, in connection with the rediscovered possibility of leaving the house and of meeting others, there was an increase of the fear of infecting others, especially family members. In fact, at t2, further fears emerged, such as the fear of the other, of going back into lockdown, of the increase in infections:

‘The fact of having to go out and meet many people on the street, who maybe are walking without a mask and without maintaining a safe distance, scares me. Then I'd rather stay at home, have my boyfriend come here and at most just go out to visit my grandmother who lives a few meters from my house’ (F, 21, Naples, t2).

The fear was also indicated by the presence of contrasting emotions: fear at t2 is mixed with joy, insecurity and awareness: ‘*I feel joy but at the same time fear for the future given the bad economic consequences of COVID‐19*’ (F, 26, Palermo, t2).

#### 4.1.3 Anger

Even regarding the area of anger, there was a sharp decline in this strong emotion in our t2. However, participants still felt anger against people who demonstrated ignorance or lack of care for the new rules established to contain the spread of the epidemic: ‘*I often see people who do not respect safe distances or who do not wear a mask*, *and all this makes me angry*’ (F, 21, Florence, t2).

Compared to the first time, however, participants no longer reported anger towards local and national institutions and towards restrictive measures imposed by them. In fact, at t1, the anger related to the limitations on freedom was prevalent: ‘*I also feel anger for the limitation on freedom of movement*’ (F, 24, Milan, t1). In the same way, anger presented itself as a response to impotence in an emergency situation far beyond one's own possibilities of understanding and action: ‘*I have experienced anger these days because this situation makes me feel helpless: a virus is an invisible enemy*’ (F, 22, Milan, t1).

At t2, we collected stories showing that anger subsides, due to the reduction of restrictions on one's personal freedom but also due to the development of greater public understanding that such restrictions were necessary to combat the spread of the virus: *‘I also felt anger when people break the rules by putting other people at risk and ‘nullifying ‘the sacrifice that many other people have made*. *The people closest to me*, *both family and friends*, *have experienced anxiety and fear*’ (F, 22, Turin, t2).

#### 4.1.4 Depressive feelings

At t1, the participants reported emotions such as impotence, nostalgia, loneliness and sadness that were indicative of the negative experience caused by social isolation: ‘*I think I reacted with an emotion of resignation in the face of losses (friends, nature, sun)*’ (F21, Genoa, t1). In the interviews at t2, that is, when the opportunity to meet others had returned, new emotions emerged, such as apathy and feeling alone with oneself, which were instead due to the fact that they were in a certain sense accustomed to the feeling of precariousness that the continuation of the emergency situation entails.

‘*At the end of the quarantine the emotions I felt were mixed, as on the one hand, I felt happiness in being able to see my friends again but also a lot of fear due to the precariousness of the situation and the possibility of returning to the starting point*’ (F, 22, Turin, t2).

Moreover, in this category, we gathered contrasting emotions. While, on the one hand, the participants reported feeling almost resigned to the state of perennial emergency in which they lived, on the other, they reported being positive about a more serene future after the resolution of the health emergency. In other words, they felt discouraged, but at the same time, they felt hope.

#### 4.1.5 Joy

Finally, a big change in participants' emotions between t1 and t2 is the appearance of a positive emotion that is joy. Compared to the lockdown, at t2, the respondents reported great joy for a partial return to normality. The participants say that they were happy for the chance to finally reunite with family and friends, to go out in the open air for walks or physical activity, and to return to their workplaces: ‘*I am happy for people who can start working again*’ (F, 20, Milan, T2). ‘*I myself have found a strong joy, as it would be possible to see the people you love again*’ (F, 22, Turin, t2).

Joy also appeared as an enhancement of what this emergency situation has allowed us to experience in terms of closeness to family and the rediscovery of important things: ‘*From this experience I take home the pleasure of being with my family*’ (F, 21, Milan, t2); ‘*Joy even for the little things*. *As soon as I left the house, I noticed a whole series of natural details that I would not have considered in the same way before*’ (F, 22, Turin, t2).

#### 4.1.6 Anxiety

Anxiety at t1 was connected to the fear of not being able to cope with such a new situation: anxiety pervades all aspects of daily life, and it was related to difficulties in falling asleep and studying and, more generally, was related to the course of normal life: ‘*In particular, the first week I lived in a state of perennial anxiety that also involved my family because the week in which the announcement happened, I started not feeling well*’ (F, 21, Genoa, t1).

The post‐lockdown time was still characterized by deep anxiety concerning the unpredictability and uncertainty of the future: ‘*a feeling of deep anxiety in waiting for what will happen to us in the coming weeks*’ (M, 21, Naples, t2).

Then, at the t2, the narratives talked about emotions and feelings concerning the reorganization of one's own life: ‘*Sometimes I felt anxious about organizing commitments in the short‐term future, not having certainty about the possibility of movement and connecting with the university*’ (F, 27, Turin, t2).

Participants described anxiety connected to the new specific circumstances they are in and they will most likely face: ‘*I think that managing this phase in a coherent and effective way is difficult, but we must keep in mind that the emergency is still ongoing and that safety measures are important*’ (F, 21 Palermo, t2).

#### 4.1.7 Post‐lockdown anxiety

Data showed a further sort of anxiety related to the specific situation of the end of the lockdown and entering a new context presumably similar to the previous one: ‘*A normality that I want and that scares me at the same time*’ (F, 26, Palermo, t2).

To put it better, narratives described anxiety related to the end of the health emergency isolation and to the restart of usual activities: ‘*I experienced intense states of anxiety related to the end of the quarantine, I felt very sad and scared at the thought of being able to leave the house again and meet other people*’ (F, 21, Turin, t2). The return to normality created disorientation and uncertainty and for some people in some circumstances brought the desire to remain isolated. In brief: ‘*there is a relief and at the same time the fear of restart*’ (F, 21, Turin, t2).

Specifically, we assumed that leaving confinement led to the loss of one's own protective shield and then exiting the house brought vulnerability and loss of personal protection:

During lockdown I had so much desire to go out and see all the people I love, but now that I finally have the chance, that urge has turned into anxiety and fear of going out. I see that people around me act as if everything is back to normal, but for me it is not so, quite the contrary. The fact of having to go out and meet many people on the street, who maybe walk without a mask and without keeping a safe distance, scares me. So, I prefer to stay at home, let my boyfriend come here and at most limit myself to going out only to visit my grandmother who lives a few meters from my house. (F, 22, Naples, t2)

### 4.2 Community dimensions

The community dimensions collected narratives related to the context, with the community and, more generally, the relationships with others outside the circle of family and friends. This macro‐category included three sub‐categories: *emotional sharing,* which had to do with the emotional and affective sharing of the emergency' experience; *connectedness,* which has to do with feeling united and the actions implemented, often using creativity and innovation, to counteract the isolation at t1 and the restrictions imposed by the anti‐COVID rules at t2; the third sub‐category was *solidarity,* which expressed the desire to help others in such a complex and risky moment, both for health and for relationships as well as the social and economic life of a country.

#### 4.2.1 Emotional sharing

In general, the sub‐categories of emotional sharing and connectedness were more prevalent at t1 of the pandemic and a bit less at t2, which was more characterized by a thoughtful look at the lockdown period that had just passed together with a certain amount of anxiety for the present and the future, especially in terms of one's relationship with the context and community. Shared distanced activities were very frequent at t1 while they are less present at t2. Lockdown was seen as a period of greater emotional sharing: ‘*We all had to stop, interrupt our lives and we lived very often with the unknown of tomorrow, but many people still thought of each other and that an appointment made and a song could keep everyone company*’ (F, 21, Florence, t1). At t2, albeit to a lesser extent, the perception of sharing the same contagion risk situation remained:

Following this situation, which was as difficult as it was unexpected, what unites the whole community are emotions and moods experienced in recent months. We have never been so similar, because inevitably, in one way or another, we all felt the anxiety and discomfort that filled our days during quarantine. (F, 20, Naples, t2).

Emotional sharing was highlighted in the acts of solidarity that united citizens engaged in a common battle: ‘*I think it is important to point out the unity between Italian citizens who are committed to helping anyone with any possible action*’ (F, 21, Florence, t2).

#### 4.2.2 Connectedness

During the lockdown, there was a considerable effort on the part of people to find alternative ways to be together: ‘*Faced with social distancing, affecting the opportunities for closer and physical relationships, alternative measures were imagined: virtual connectedness, a sense of connection expressed by shared rituals (playing, singing, toasting …), organization of community and neighborhood support aimed, first of all, at the most fragile citizens*’ (F, 23, Milan, t1).

Throughout all of Italy, the most visible and usual way to strengthen connections among citizens was certainly the daily appointment on balconies to sing together: ‘*A beautiful and positive action that was implemented is singing at 6:00 pm from balconies of the houses, an act of solidarity and community*. *And also, the applause of doctors and nurses at 12:00*’(F, 21, Palermo, t1). ‘*In such a difficult moment, we support each other, the whole world supports each other, through moments of prayer, through the flash mobs that are organized to show solidarity and unity from the balconies of their homes*’ (F, 20, Palermo). It is evident that what, in the lockdown phase, was an expression of connectedness in order to feel less alone was re‐signified during the post‐lockdown as an experience of collective solidarity:

Personally, I found the phenomenon of concerts from the balconies that were repeated almost daily in the first periods of quarantine really beautiful; it reminded me that ‘no man is an island’ and when he is forced to be, he will spontaneously seek an alternative way to anchor himself to others. (F, 21, Florence, t2).

In the post‐lockdown, there was also the awareness that we need to do more, to go beyond flash mobs and work to create connections, not only in the moment of maximum difficulty, but also later so that in difficult situations (at least on a superficial level), there was more connection: ‘*And it would be nice if this did not come out only with flash mobs or similar events, but that in reality in everyday life, that people manage to be an active part in the life of society*’ (F, 23, Turin, t2). The desire for greater stability and duration of the actions implemented in an extemporaneous form during the lockdown gained ground among citizens:

It would be nice in this circumstance for the region to create associations to promote social connection, with activities to be done either at a distance or by dividing people who wish to do them into different hours and activities. This could be a nice way to make people flourish after this stressful event, and make them understand that they are not alone, that in moments of difficulty one can react. (F, 23, Turin, t2).

#### 4.2.3 Solidarity

If at t1 the gestures of solidarity described by the participants referred to the present moment of isolation: ‘*I very much appreciated that several restaurants, during the lockdown, did not decide to throw away food, but to cook it and offer it to hospitals*’ (Genoa, F22, t1), in the narratives of the participants at t2, a description of solidarity actions emerged, which mainly refer, however, to the past: ‘*In the village of a friend of mine, those who went down to the city knocked on the door of their fellow villagers to ask if they needed anything*’ (Genoa, F23, t2). During the lockdown, solidarity concerned all citizens and was very much linked to the desire for connection among citizens; conversely, when solidarity gestures were described during the post‐lockdown, it was worth to note that they were directed more specifically towards the weakest sections of the population, those who have been most damaged by the pandemic. Above all, solidarity had poor families as a preferential target through the distribution of food.

A positive action to which due importance must be given in my opinion is the spread of <suspended spending> in this period of crisis, a way to help out by paying for basic necessities for people who found themselves in difficulty with the closure of all activities or those who were already before the emergency, which contributed to aggravating the situation of many people. (F, 20, Naples, t2).

Then, at t1, solidarity action originated from the ordinary citizen involved in their personal capacity, as well as described by a previous quotation; likewise, at t2, specialized personnel was called to actions of solidarity based on their political or professional function: ‘*My focus is the social initiative implemented by associations and public figures, in order to support families and citizens in difficulty*’ (F, 20, Naples, t2).

The value of the voluntary actions implemented both during the lockdown and in the subsequent t1 was recognized: ‘*It seems important to me to pay attention to all those who have carried out even small actions of support and help, especially towards elderly and needy people, and who still continue to do so*’ (22, F, Turin, t2).

## 5 DISCUSSION

The results described indicate a change between t1 and t2 involving, above all, the meaning given to events and to one's own behavioural reactions. The emotions of the two times remain substantially the same but are expressed towards different objects: at t1, anxiety, fear, but also anger, address the forced social restrictions and the related reorganization of one's daily routine. At t2, joy for gaining new spaces of freedom appears but, at the same time, anxiety and fear of a return to normality full of uncertainties and risks remains.

As far as the community dimensions are concerned, the main difference is their decrease from t1 to t2. There is also change in the way collective behaviours of emotional sharing, connectedness and participation are implemented, from an initial reactive push that led the participants to feel very involved, precisely in response to isolation, to a greater awareness of the importance of organized and punctual action. It is interesting to note that it is the semantic dimension attributed to the individual and community dimensions that changes, and not so much their nature.

From the macro‐categories we proposed, it is evident that, in the emergency distancing phase, solidarity reaches levels that we could call almost heroic. Connectedness was at its highest level, as literature defined it, as a sort of collective honeymoon (Kaniasty & Norris, 2004). At the boundary between t1 and t2, anxiety was also increasing as well as a sort of ambivalence between acting as if nothing new is happening and new, unexpected and unspeakable worries. At the same time, there was a new awareness that ‘staying at home is not so bad’. People rediscover family bonds, the pleasure of being in their own space, and there are some interesting reflections about the previous experience of always being out and in perpetual motion from place to place.

Therefore, anxiety for the future, disorientation, worry about how others will behave are sometimes accompanied by a more subjective feeling, not related to a specific object, that we called post‐lockdown anxiety. As Iorio, Sommantico, and Parrello (2020) said, the COVID‐19 pandemic is associated with increased psychological distress, depression and anxiety, specifically post‐lockdown anxiety, as it was evident in the first days after the lockdown (Disillusionment phase). Therefore, the anxiety experienced by the participants does not undergo a quantitative but rather a qualitative change: the content and form taken by the anxiety were transformed. If, at t1, it is an emotion connected above all to isolation, in t2, it is the uncertainty that elicits the anxiety response.

Moreover, there is acknowledgement that in the post‐lockdown collective feelings and action were reduced. Anxious emotions took over and the community dimensions took on more specific and coordinated forms, less impromptu, and community was mostly considered a problem by individuals. As stated in other contributions (Di Napoli et al., 2021; Migliorini et al., 2021) in the first phase of emergency, strong feelings of fear (Pulcini, 2001), together with the awareness of being united with other human beings as well as intense perception of vulnerability and weakness, induced people to renew the desire for bonding, generating and reinvigorating their desire for community (Marta, Marzana, Aresi, & Pozzi, 2016). In the following phase, the drive for sociality and solidarity was more ambiguous, and the personal behavioural responses were more varied, also due to anxiety. In fact, as the duration of the emergency and the related perception of risk increase, anxiety and uncertainty also increased.

Despite the fatigue linked to the restrictions, the lockdown allowed shared emotional and behavioural responses and, therefore, was most reassuring; conversely, during post‐lockdown, the perception of uncertainty and risk increased, the behavioural and emotional responses became more fragmented, and the anxiety took on more pronounced traits (post‐lockdown anxiety).

Several limitations of the current study suggest possibilities for future research. First, our research focuses on Italian university students, and it would be interesting to have data concerning the whole youth population, including those from different countries, to generalize the results. Second, a sample composed specifically of students limits the possibility of generalizing the results. Third, the lack of a third data collection time prevents us from exploring further phases of this emergency. At the time, we collected the data, it was not possible to predict the development of the pandemic. We believe that a data collection, even with a different sample, could equally offer reflections on how emotions, coping strategies and relationships with the community are developing, evolving over time. In fact, the reconstruction phase is not yet discernible, and it would be interesting to see how personal emotions and community dimensions change with the cyclic recurrence of the emergency phases.

## 6 CONCLUSION

In the first lockdown, feelings of connectedness were widespread; in the following post‐lockdown, there was some evidence of positive emotions (joy), but also an increase of ambivalent emotions. In some way, the lockdown promoted a shared connectedness; meanwhile, the ‘return to normality’ reintroduced an individual perspective on problem solving. The results showed that the trend of the pandemic is somehow like the first and second phases of an emergency: the *Heroic phase* and the *Honeymoon phase*. In the first one, strong emotions were evident and people were forced to carry out heroic actions, in the second phase the whole community was sharing the difficult experience and people tried to unite overcome the emergency situation. These two phases activate everyone, and emotions played a role as motivator of social actions. The community dimensions were very accentuated in these phases.

In phase 3 (*Phase of Disillusionment*), conversely the emotions of anger and resentment prevailed, the sense of community sharing was gradually lost, and people started to focus on personal problems in order to return to normality. The results of the present work detected also the increasing of a disillusionment impact; emotional ambivalence appeared: fear and joy, as well as the coexistence of anxiety and joy. The community dimensions underwent changes both in intensity and in the form in which they were expressed.

Moreover, in a community psychology approach, Procentese and Gatti (2019) highlighted the importance of social awareness and responsibility. In their view, social distancing cannot be a social obligation; it should be a shared goal that citizens pursue in a spirit of social conviviality and connectedness. In this sense, social regulations and directives must be chosen according to individual needs and health measures. This is a significant social conundrum where community psychologists can help in promoting measures for social awareness and public understanding. The specificity of this emergency leads us to evaluate what is happening by referring to different levels of analysis: individual, family and relational dimensions, but also those of the local and global community. In this vein, our research will contribute to promote adequate social measures to contrast the pandemic effects, but also the impact of the health distancing measures especially on specific groups as students, and their potential distress (Zurlo, Cattaneo Della Volta, & Vallone, 2020).

In future studies, it would be valuable to investigate what coping strategies people used to manage the emotions they experienced. Furthermore, it might be interesting to investigate the evolution of people's sense of responsibility and their involvement in pro‐social and solidarity actions. In operational terms, it would be useful to work on the development and stabilization of people's social responsibility, which not only increases the sense of community but also solidarity: in other words, it generates resilient communities. The link between responsibility and being brothers is therefore close. Being responsible means being able to respond: responsible action always translates into an answer to a question that comes from the other and results in solidarity.

## CONFLICT OF INTEREST

The authors declared no potential conflicts of interest with respect to the research, authorship, and/or publication of this article.

## ETHICS APPROVAL

The research was approved by the Ethical Committee of the Department of Humanities at the University Federico (Cerp, Ethics Board for Research in Psychology, March 15, 2020).

## Supporting information


**Data S1.** Supporting information.

## Data Availability

The data that support the findings of this study are available from the corresponding author upon reasonable request.
