# Supplementary material for: Community dimensions and emotions in the era of COVID‐19
Source: J Community Appl Soc Psychol. 2021 Aug 15;32(3):358–73. doi: 10.1002/casp.2560 (PMC8427116; doi:10.1002/casp.2560)
Supplement: Supplementary file 1 — Data S1. Supporting information. [file CASP-32-358-s001.pdf]

Community Dimensions and Emotions in the Era of COVID-19

AUTHORS:

Marzana D., Novara C.,De Piccoli N., Cardinali P., Migliorini L., Di Napoli I., Guidi E., Fedi A., Rollero C., Agueli B., Esposito C., Marta E., González Leone F., Guazzini A., Meringolo P., Arcidiacono C., Procentese F.

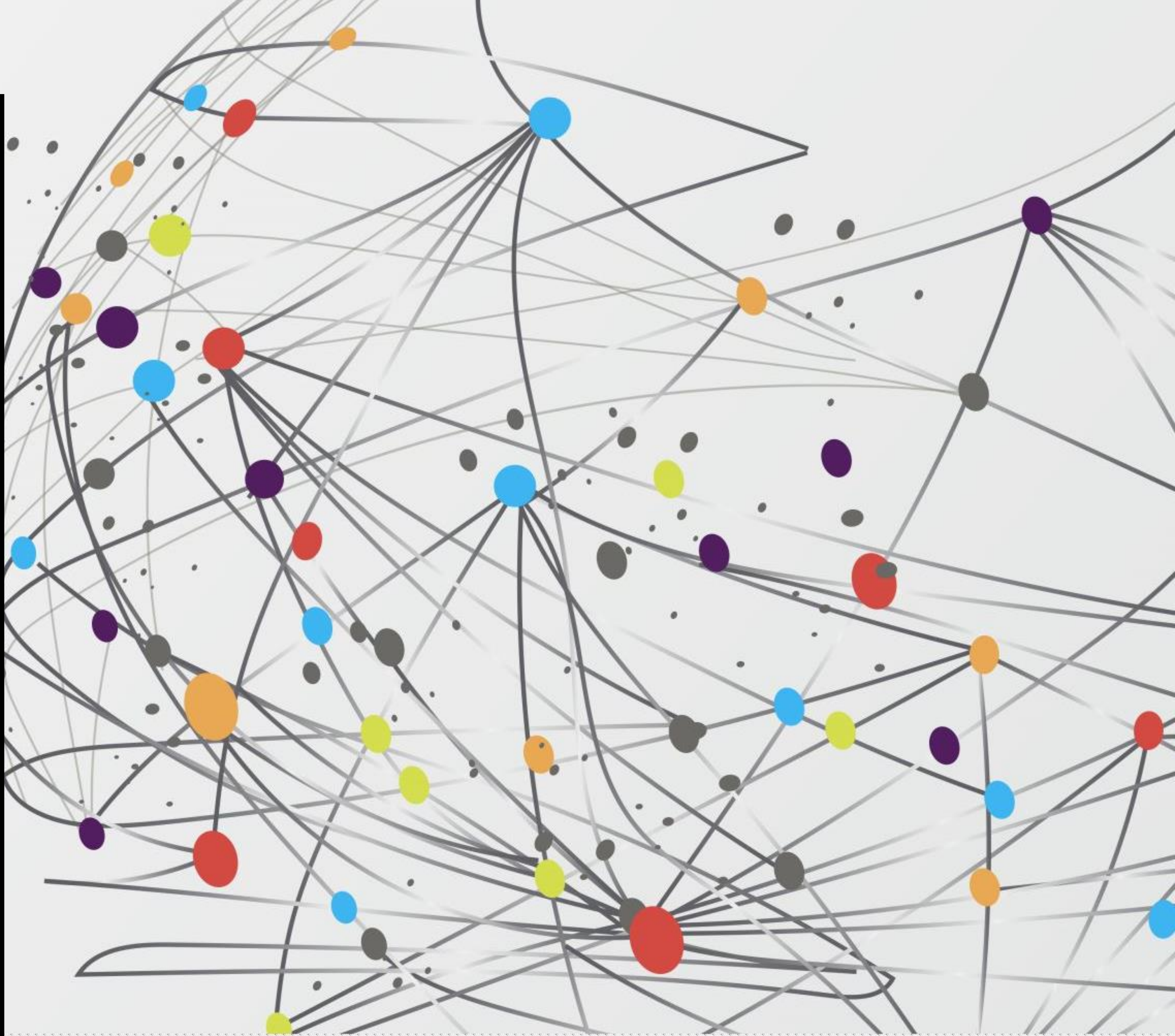

Following an ecological perspective, reactions to a disaster – such as the COVID-19 pandemic - should be analyzed in the interdependence between individual and community dimensions.

- The COVID-19 pandemic and the correlated restrictions have had a tragic impact on the lives of people and communities; for this reason, it becomes important to understand individual emotional responses and their community dimensions with respect to this devastating event.

The AIM of the study was to explore the behaviors, feelings, thoughts and actions of university students during the lockdown and immediately after. Specifically, we were interested in probing the inner world of young people confronted with this unexpected event.

The two dimensions on which we focused are:

(a) the **individual emotional dimension**, experienced over time in changing conditions, and

(b) **community dimensions** such as connectedness, emotional sharing and solidarity, which can emerge in emergency situations such as that produced by COVID-19.

746 participants (T1- March 2020)
361 participants (T2- May 2020)
with a mean age of 21.93 years

Some evidences from the research…

Individual Emotional Dimension

- The emotions during the lockdown (t1) and after (post lockdown- t2) remain substantially the same but are expressed towards different objects: at t1, anxiety, fear, but also anger, address the forced social restrictions and the related reorganization of one's daily routine. At t2, joy for gaining new spaces of freedom appears but, at the same time, anxiety and fear of a return to normality full of uncertainties and risks.
- Anxiety for the future, disorientation, worry about how others will behave are sometimes accompanied by a more subjective feeling, not related to a specific object, that we called **post-lockdown anxiety**

Community Dimensions

- There is also change in the way collective behaviors of emotional sharing, connectedness and participation are implemented, from an initial reactive push that led the participants to feel very involved, precisely in response to isolation, to a greater awareness of the importance of organized and punctual action.
- The lockdown promoted a shared connectedness; meanwhile, the ‘return to normality’ reintroduced an individual perspective on problem solving.

In conclusion

Social distancing cannot be a social obligation; it should be a shared goal that citizens pursue in a spirit of social conviviality and connectedness. In this respect, social regulations and directives must be chosen according to individual needs and health measures. This is a social conundrum where community psychologists can help in promoting measures for social awareness and public understanding. The specificity of this emergency leads us to evaluate what is happening by referring to different levels of analysis: individual, family and relational dimensions, but also those of the local and global community.
